# Muslim women’s knowledge, views, and attitudes towards sexually transmitted infections in Saudi Arabia: A qualitative study

**DOI:** 10.1371/journal.pone.0286822

**Published:** 2023-06-23

**Authors:** Noura Alomair, Samah Alageel, Nathan Davies, Julia V. Bailey

**Affiliations:** 1 Community Health Sciences Department, College of Applied Medical Sciences, King Saud University, Riyadh, Kingdom of Saudi Arabia; 2 Research Department of Primary Care and Population Health, Institute of Epidemiology and Health Care, University College London, London, United Kingdom; National Institute of Public Health: Instituto Nacional de Salud Publica, MEXICO

## Abstract

**Background:**

The cultural sensitivity surrounding sexuality in Islamic communities has an impact on awareness and prevention of sexually transmitted infections (STIs). This study explores Muslim women’s knowledge, views, and attitudes towards STIs and people living with HIV/AIDs in Saudi Arabia.

**Methods:**

We conducted qualitative semi-structured interviews with Muslim women from Saudi Arabia. Interviews took place in a public hospital in Riyadh, Saudi Arabia in 2019. Data were transcribed, coded, and analysed using a reflexive thematic analysis.

**Results:**

Twenty-eight women were interviewed, the majority were college educated and employed. Participants lacked knowledge about STIs, and there were significant misconceptions. The majority of women expressed extremely negative attitudes towards STIs, particularly towards people living with HIV/AIDS. Participants believed that judgemental attitudes and stigmatisation of people with HIV/AIDS were justified if an infection was transmitted through extramarital sex. Men were believed to be the source of STIs, and STIs were viewed as punishment from God for extramarital sexual relations that are forbidden in Islam. Protection against STIs was believed to be achieved by strengthening religious beliefs.

**Conclusion:**

Attitudes towards people with STIs, HIV/AIDS in particular, were highly influenced by religious views towards extramarital sex, as well as lack of knowledge and misconceptions. There is an urgent need for accurate information and improved awareness of sexual health including STIs among Muslims in Saudi Arabia. Public health efforts should be directed towards reducing stigma and discrimination against people living with HIV/AIDS in Saudi Arabia and other Islamic communities.

## Introduction

Sexually transmitted infections (STIs), including human immunodeficiency virus/acquired immunodeficiency syndrome (HIV/AIDS), continue to be a major public health issue globally. The rates of STIs vary across geographical regions, and the global incidence of HIV/AIDS declined in the last decade [1]. However, an increase in HIV/AIDS and other STIs in the Middle East and North Africa (MENA) region have been documented in recent years despite evidence suggesting that these rates are underreported [2]. Religious, cultural and social taboos are a barrier to the collection and reporting of accurate data on STIs prevalence and incidence in the MENA region [2]. Reported attributable risk factors for STIs include condomless sex, injecting drug use, and intimate partner violence [1].

The cultural sensitivity surrounding sexuality in Islamic communities has an impact on STIs awareness and prevention. Religious and cultural factors are a major barrier for Muslims to acquiring STIs information and services [3, 4]. This poses a significant challenge, especially since religious views can impact on Muslims’ perceptions of their susceptibility to STIs and the need for sexual health information [4, 5]. Many Muslims believe that STIs are uncommon among them as religious beliefs offer sufficient protection against STIs [4].

Muslims’ attitudes towards STIs, and HIV/AIDS in particular, are highly influenced by lack of knowledge and misconceptions [5, 6]. Several studies among Muslims in different settings worldwide report people advocating for the isolation of people living with HIV/AIDS from the community [5–7]. The extreme negative views towards people living with HIV/AIDS significantly affects their access to medical care. People living with HIV/AIDS report being refused medical treatment and discriminatory behaviour from medical staff [8].

Several studies from Saudi Arabia suggest a lack of STI knowledge and extreme negative views towards HIV/AIDS [9–11]. Lack of knowledge about STIs modes of transmission and ways of prevention contribute greatly to fear of people living with HIV/AIDS [9–11]. Negative attitudes and misconceptions were also common among physicians and other healthcare providers in Saudi Arabia [10, 11].

There has been an increase in the rates of STIs in Saudi Arabia in the last decade [12]. In 2020, there were approximately 12,000 people known to be living with HIV/AIDS in Saudi Arabia compared to 4,800 in 2010, with the majority of the infections among men [12]. Women are especially vulnerable as most risk behaviours (i.e., infidelity and injecting drug use) in the MENA region are practiced by men [13]. Sexual activity in Muslim communities is highly influenced by gender roles, and marriage is reported as the main route of HIV transmission for Muslim women [13–16]. Women in the MENA region are also at a higher risk of under-detection of HIV/AIDS and other STIs [17]. Reports from the MENA region suggest that some healthcare providers misrepresent and/or hide STI information from women, making Muslim women particularly vulnerable to STIs [18].

Few studies have examined the role of cultural, social, and religious factors on Muslim’s attitudes towards STIs [9, 19–21]. All existing evidence examining STIs and HIV/AIDS attitudes and beliefs in Saudi Arabia used quantitative research methods. The sensitivity of the topic and the complexity of the issue requires qualitative research methods to provide in-depth understandings. The aim of this study was therefore to explore Muslim women’s knowledge, views, and attitudes towards STIs and people living with HIV/AIDs in Saudi Arabia.

## Methods

### Study design

This study used qualitative research methods with semi-structured interviews. Data were collected as part of a larger research project, exploring multiple aspects of the sexual and reproductive health of Muslim women in Saudi Arabia [22, 23]. The research was guided by the conceptual framework produced by systematic reviews of the literature on the complex set of factors influencing Muslim women’s sexual and reproductive health [4, 5]. The framework was drawn from a modified version of the ecological model of health behaviour [24]. The conceptual framework provides an explicit consideration of multiple levels of factors influencing women’s experiences and views, including religious, cultural, social, and personal factors.

### Sample

Participants were purposively sampled, recruiting Saudi women aged 18–50, sampled by age, marital status, educational level, and employment. This heterogeneous sample allowed for the capturing of a wide variety of views and experiences. Recruitment was conducted in a public hospital in Riyadh, Saudi Arabia from January to June 2019.

### Data collection

Interviews took place in Riyadh, the capital city of Saudi Arabia, in a public hospital, King Fahad Medical City (KFMC) in a private room in the women’s health clinic. The recruitment process was done by the lead author (NA), between January and June 2019. This hospital is one of the major public hospitals in Riyadh that provides primary, secondary, and tertiary healthcare services to the public free of charge.

Potential participants were approached in the waiting areas of the hospital’s outpatient clinics and invited to take part in the study. An information sheet was handed to potential participants where the research aims, and research questions were explained and any questions or concerned raised by participants were addressed prior to the interviews. Written consent was obtained before the start of each interview. Interviews were audio-recorded with participants’ permission. All interviews were conducted by the lead author, a Saudi female public health researcher with experience in qualitative research. The interviewer had no established relationship with any of the participants before the commencement of the study. The interviews were conducted face-to-face, ranging between 30 to 90 minutes. Recruitment continued until data saturation was reached. Participants filled in a demographic questionnaire to obtain data on age, marital status, parity, educational level, and employment.

The topic guide explored women’s knowledge, views, and attitudes towards STIs. Some questions in the topic guide relate to sexual health, which can be considered a sensitive subject to some participants. It is considered unacceptable in Muslim cultures to assume that unmarried women are sexually active, as this could be construed as accusing participants of having premarital sex, which can have severe repercussions for unmarried women. As a result, questions were phrased differently based on marital status to avoid offending participants. The guide was piloted with a few participants and questions were rearranged and rephrased based on their comments. The piloted interviews were not included in the analysis as the purpose was to improve the topic guide.

### Data analysis

The audio-recorded interviews were transcribed verbatim and data were analysed using reflexive thematic analysis [25]. A reflexive thematic analysis offers theoretical flexibility in answering broad questions about people’s experiences, perceptions, and their representations of a particular phenomenon. Reflexive thematic analysis acknowledges that all analyses are influenced by the researcher’s positionality. All interviews were conducted and analysed in Arabic. A selection of interviews was translated to English for non-Arabic speaking members of the research team to discuss findings. Interviews were coded line-by-line by NA using ATLAS.ti software. A random sample of interviews were coded by another member of the research team (SA). Data was analysed in an inductive approach, where themes and codes were generated from the data using coding and refinement of themes. Codes from each transcript were compared, discussed, and where needed, amendments were made, until agreement among authors was reached. Codes were grouped together producing an analytical framework and the preliminary themes were created. The analytical framework was refined in an iterative way throughout the analysis via discussions with all authors. Quotations to support the findings were translated into English for reporting.

### Ethical approval

The study was approved by UCL ethics committee (Reference no. 10157/001). Ethical approval from the hosting hospital in Riyadh was obtained on 30/01/2019, Reference no. FWA00018774.

### Findings

Twenty-eight women, aged between 20 to 50 years, participated in the study. Most participants had a bachelor’s degree and were employed. More than half of the sample (n = 16) were married. **Table 1** provides an overview of the sample’s characteristics.

**Table 1:** Key characteristics of study participants.

**Marital status**	**N**
** Married**	16
** Single**	9
** Divorced**	3
**Age**	**N**
** 20–25**	7
** 26–30**	3
** 31–35**	8
** 36–40**	7
** 41–50**	3
**Number of children**	**N**
** 0**	14
** 1–2**	2
** 3–5**	12
** +6**	1
**Education**	**N**
** Bachelor degree**	26
** Diploma**	2
**Employment**	**N**
** Employed**	18
** Unemployed**	5
** Student**	5

### Sexually transmitted infection knowledge

Most women lacked knowledge of STIs, mainly mentioning AIDS when they were asked about STIs they had heard of. Participants did not generally understand the difference between HIV and AIDS. Women with a college education in medical sciences had heard of syphilis and less commonly mentioned gonorrhoea and herpes. These four STIs were the only STIs mentioned by participants.

“*The only one I know is AIDS*, *maybe UTIs*. *But they told me this is not an STI*. *Even if it’s a bladder infection*, *or something like that*, *I heard it’s not transmissible*. *It’s not related to sexual intercourse*. *So*, *I only know of AIDS*.*”* **(P11, Divorced, 30 years old)**

Women held misconceptions about the mode of transmission of STIs and possible treatments. Almost all women thought that HIV is transmitted by sharing personal items, like clothing and using the same utensils.

"*If I’m not mistaken syphilis is through sex but AIDS no*, *it can be transmitted through using personal items like the spoon of infected individuals meaning through saliva*, *or sneezing*. *I don’t know if my fear [of people living with HIV] is irrational*, *I am not educated enough on how to protect myself from such diseases*, *so I don’t want to deal with them [people living with HIV]*.*"* **(P19, Married, 38 years old)**

Women were aware of their lack of knowledge when it comes to STIs. This was evident in women’s answers, with some making jokes about their ignorance while others were critical about their lack of knowledge.

“*AIDS*, *herpes and syphilis*, *but how did I hear about it*? *That’s the question*. *Not any reliable source*. *I mean if it wasn’t for personal efforts and research I wouldn’t know*, *as far as formal education goes*, *nothing credible*. *Even in university it wasn’t fully covered*.*”* **(P8, Single, 28 years old)**

### Sexually transmitted infections are “not our problem”

Women tended to use phrases like *‘God forbid’* and *‘God forgive us’* before mentioning STIs. When asked if they think that STIs are common in Saudi Arabia, the majority of women in the sample believed STIs are non-existent in ‘*our country*’.

**“*NA*:** *Do you think STIs are common here in Saudi*?

***P7*:** *No thank God*, *we don’t have it here in Saudi because extramarital relations are forbidden*, *and we have our religious beliefs to protect us*.*”* **(P7, Married, 44 years old)**

Religion was viewed as the most dominant influence on women’s views towards extramarital relations. Women believed that God fearing Muslims do not engage in extramarital sex, and therefore did not perceive STIs to exist in the country. Social norms were a significant influence. For example, one participant explained that even when people are not *‘religious’* and are *‘open-minded’*, they will not practice extramarital sex because it is frowned upon in Saudi society. When this participant was asked why she thinks STIs are not common in Saudi Arabia, she said:

“*Maybe because religion prevents forbidden relations*, *which are the cause of these things [STIs]*. *But*, *other than religion*, *some people could be open-minded*, *and they don’t care if it’s forbidden*, *but because of our traditions and the fact that it is socially unacceptable to have extramarital relations people won’t do it*. *And that might be the reason why STIs are not common here*.*”* **(P27, Single, 22 years old)**

Some women acknowledged that extramarital relations happen and believed that STIs exist but said that they cannot comment on the magnitude of the problem with certainty due to lack of publicly available data. Participants also raised the issue of transparency and openness in discussion relating to STIs in the country.

“*It’s not that prevalent*, *but we have it*. *Facts are hidden*. *There is no transparency*. *Data is not publicly available*, *and we know we can’t talk about it*.*”* **(P8, Single, 22 years old)**

Linking lack of religious morals with having an STI led women to believe that these diseases could be an issue in the future due to recent societal openness. In recent years, with technological advances and the advent of social media, Saudi society has been exposed to different cultures, and gaining access to information has become easier. Some women viewed this as a negative change influencing young people’s minds and *‘opening their eyes’*. Discussion and interaction between genders is becoming easier and more acceptable in Saudi culture, which led some women to conclude that this was one of the reasons why STIs are becoming more prevalent.

“*Society is more open now; a lot of things became acceptable*. *Not everyone used to be able to travel before*, *now everyone travels*. *I am talking about when I was young*. *Now*, *it is different*. *The majority are travelling and exposed to different cultures*.*”* **(P20, Divorced, 40 years old)**

Women often used the term *‘travelling’* as a code referring to extramarital sex and *‘forbidden relations’*. This also stems from the belief that extramarital sex and STIs do not exist in Saudi Arabia since it is a Muslim country but are *‘problems’* that *‘foreigners’* or Saudi men bring back home from other countries.

“*No thank God*, *these diseases are not seen in my social circle and environment*. *I hear foreigners who come here to work bring such diseases*. *But in my social circle*, *thank God it does not exist*.*”* **(P3, Married, 33 years old)**

“*What I usually hear is that the man did something forbidden*, *he travels to a bad country and gets an STI and gives it to his wife… I feel it is mostly men*, *given that men are drawn to these things [sex] more*. *But infected females are unheard of*.*”* **(P9, Single, 22 years old)**

### Men as the source of infection

Participants placed the blame on men for women acquiring an STI. Women with an STI are usually seen as victims of the husband’s infidelity and unfaithfulness, rarely being mentioned as having an STI from any source other than her spouse. Either he had an STI before getting married or being unfaithful during their marriage. There was a sense of acceptance over men having extramarital sex and women are told to *‘just be careful’* when their husbands travel abroad, as travelling is also seen as a gateway to having extramarital sex.

“*Any woman has to be careful and take precautions when her husband comes home from travelling*. *Even if he was a Shaikh [Religious leader]*, *she should avoid him for a while*, *she has to protect herself*.*”* **(P7, Married, 44 years old)**

All women mentioned lack of religious morals and having no fear of God as one of the causes of STIs, which woman often ascribed to men. Women explained why men are more susceptible to STIs by giving reasons like men do not have to fear the consequences as women do and have the freedom to travel and go out freely without needing permission from a guardian or a parent. These beliefs were generally deep rooted and accepted as immutable.

“*Because we live in a male dominant society*. *Because men have no culturally set boundaries like women do*. *There is also the fact that males are not like females*. *Females have virginity*, *so it shows*. *Whereas the male has no prohibitions*. *No one can know*. *So*, *it is easier if you try it [sex] before [marriage]*.*”* **(P8, Single, 28 years old)**

### Protection against sexually transmitted infections

Prevention of STIs was believed to be achieved through strengthening religious beliefs and having a solid ‘moral compass’. Only one participant mentioned condoms as a method of protection against STIs. Many women emphasised the importance of raising awareness about STIs in the context of educating women so that they know what symptoms to look out for if their husbands are unfaithful or acquired an STI before marriage, confirming the narrative of men being the source of infection.

“*The disaster is that most men have premarital sex*, *and then the poor woman is the victim when she gets married*, *because she is confident in herself and that she would never get involved in premarital sexual relations*. *But then discovers that he is*, *he has been playing around and he infects her*, *but what’s even sadder is that she doesn’t know what kind of infections she could get*, *how it is transmitted and what symptoms to look out for*.*”* **(P8, Single, 28 years old)**

A few participants suggested exaggerating the consequences of having extramarital sex as a way of preventing STIs.

“*We have to raise awareness but emphasise that education should use fear appeal and exaggerate this thing’s [STI] complications*. *Yes*, *yes using fear*. *For example*, *they could make a short film and show how a person’s life became hell after [having sex]*. *This would make a stronger impact*. *Of course*, *this comes after urging people to stay in the path of God and doing it the Halal way through marriage*.*”* **(P3, Married, 33 years old)**

### Attitudes towards people with sexually transmitted infections –“it’s a punishment from God”

Most participants held negative attitudes towards individuals with an STI. Some women had judgmental views towards people with an STI, using phrases like *‘he caused this to himself’*, and *‘it’s a punishment from God’*. It is worth noting that they often used gender-specific pronouns (he/him) when speaking about someone with an STI. In other instances, it was clear in some women’s initial responses that they were conscious about seeming judgmental and tried to convey more tolerant views towards people with an STI. However, the more they talked, the clearer it became that they held very negative views.

“*Do I judge the person*? *No*, *because for some people it is out of their hands*. *It is true that they’ve got it*. *And I am not condoning Haram [forbidden] relationships but sometimes she is married but she got it from her partner*. *So no*, *no I don’t judge like that*. *I am only judging health-wise*.*”* **(P8, Single, 28 years old)**

“*I don’t think it is their fault*, *because God already punished them*. *I will try to distance myself*, *and an antiseptic will always be with me*. *So that indirectly*, *not being obvious about it*, *I would sanitize my hand after touching anything they touch*, *but in a non-obvious way*. *Because after all they are only human*. *I don’t want to hurt their feelings*.*”* **(P3, Married, 33 years old)**

People with an STI are often viewed as bad people who committed *‘immoral’*, *‘illegal’*, and *‘forbidden’* actions. Participants had extremely negative views towards people living with HIV/AIDS in particular. This could be attributed to the way HIV/AIDS is viewed by society and how it is depicted in the media, specifically in movies and TV shows. This has also contributed greatly to the misconceptions about the infection, its causes, symptoms, and modes of transmission. One of the reasons women gave for their fear and extreme negative views towards people with HIV/AIDS compared to other STIs, was the fact that HIV/AIDS has no cure and is often viewed as a terminal illness.

“*Sadly*, *I am one of those people who would be scared of people with AIDS*… *I can’t explain my reasons*, *and I don’t have valid reasons for that matter*. *But we grew up being taught that AIDS is a scary disease that could spread through the air*. *I know that there should be sexual contact*, *but we were fed from a very young age*, *not only from society or family*, *but also from the media [TV shows and movies]*. *AIDS means that this person should be isolated from the public*. *People shouldn’t be in contact with them*, *people are scared of them*. *And that a person with AIDS is a bad bad bad bad bad person*. *You shouldn’t even know someone with AIDS*. *Even though some people get it by accident*, *it doesn’t have to be through sexual contact*. *But I cannot be associated with someone who has AIDS*. *I could know someone who has syphilis or gonorrhoea*, *but not AIDS*. *Because society did not stigmatise it*, *made it a huge issue and planted fear of it like AIDS*, *AIDS is something else*…*”* **(P23, Married, 29 years old)**

### Attitudes towards people with a sexually transmitted infection—“depends on how they get it”

The first response many of the women gave when they were asked about their views towards people with an STI, was *‘it depends on how they got it’*. For example, if it was from extramarital sex, they would not be as tolerant and accepting of them. Whereas if it was from something that is *‘not their fault’* such as blood transfusion, they might be more tolerant towards that person.

“*It depends*. *If he caught it through blood transfusion*, *and it has nothing to do with travelling abroad*, *he’s not playing around*. *For example*, *like the kids in Jizan*, *from where they got it from*? *Is it blood transfusion*? *They are innocent kids*, *they know nothing*. *So It depends on the case… It depends on the mode of transmission*. *If he’s someone who plays around*, *then honestly no*, *I wouldn’t accept living with him*.*”* **(P5, Married, 43 years old)**

Reasons behind fear of people living with HIV/AIDS are at times linked to lack of knowledge and misconceptions about modes of transmission. One woman explained that she was extremely uncomfortable interacting with a person with HIV/AIDS at work. She attributed this unease to lack of knowledge about the infection.

“*I mean it’s a bit scary [HIV/AIDS]*. *And when I didn’t know*, *back then I used to think it could be transmitted even by shaking hands*. *It used to really scare me*. *But now that I have an idea … Once we had a patient with AIDS and I was confused about how they would let her in a clinic like our clinic*. *And then*, *I did my research*, *and I found out that it cannot be transmitted by hand shaking*. *And I felt at ease*. *I was like ok I’m going to be ok*.*”* **(P27, Single, 22 years old)**

### Views towards a spouse with a sexually transmitted infection

Women had conflicting views towards having a spouse with an STI. When discussing a hypothetical scenario for unmarried women, they were extremely against living with a spouse who has an STI and said that they would never accept his infidelity. Some married women explained that they would not forgive their husbands for being unfaithful and contracting an STI but said they would stay in the marriage for the sake of their children. Stigma surrounding divorce and how divorced women are perceived in society could be another factor that would force women to stay in the marriage.

“*It depends on the kind of husband he is*. *I’ve never been through this*, *and God forbid I wouldn’t*, *if it was just one time*, *I might look the other way*. *But I would isolate myself from him completely*, *I will just live for my kids*, *so that they would grow up with a mother and a father in the same household*.*”* **(P14, Married, 38 years old)**

“*What I am sure of is that I would never start a relationship with someone who has an STI*. *But if it was someone else like at work*, *it might be okay*. *But to be honest with you I would still be careful with them*.*”* **(P13, Single, 23 years old)**

When women were asked about a husband’s views towards having a wife with an STI, they explained that it would be impossible for any Muslim man to accept living with a woman who has an STI. There was an acceptance of this gender inequality, linking it with religion and how God made males and females different. It was noticed that this belief makes women feel guilty for not accepting *‘men’s nature’*, indicating that it would be going against *‘our values’* as Muslims and God’s creation of each gender. When asked if a man could live with a woman who has an STI, one woman said:

“***P14*:** *Impossible*, *I swear it would be impossible*.

**NA:** why?

***P14*:** *That’s how they are*. *God made them this way*. *We can’t change how they think*, *or how their brains work*. *Even if you tried*, *it wouldn’t work*. *It’s not like women*, *women are givers by their nature*. *But men*, *that’s how they are*, *they just take*. *God made us this way; we can’t say anything about it*.*”* **(P14, Married, 38 years old)**

## Discussion

Women in the study had poor knowledge of STIs and misconceptions regarding modes of transmission. Extremely negative views towards people living with HIV/AIDS were voiced by all women interviewed. The stigma and negative views were highly influenced by lack of knowledge, social norms and religious views towards extramarital sex. Judgement towards people with an STI was influenced by route of transmission (i.e., extramarital sex); and STIs were viewed as God’s punishment for sinful behaviours.

Women in this study exhibited extremely negative views towards people with an STI. This was consistent with existing evidence on Muslims’ attitudes towards people with any STI, more specifically HIV/AIDS [5]. Attitudes towards people living with HIV/AIDS are influenced by many factors including lack of knowledge, religious beliefs and social norms [5]. Often at times, the rejection and stigma around people living with HIV/AIDS are highly influenced by social disapproval of the acts that lead to the infection. Many Muslims associate HIV/AIDS with sex outside of marriage, sex work, drug use, or homosexuality [13, 26], all of which are considered sinful and immoral acts in Islam that some believe are punishable by death [27]. Nonetheless, stigma and discrimination against people living with HIV have been consistently reported worldwide. Evidence from Australia, Europe and United States show HIV-related stigma from healthcare workers and the general population [28–30].

Women in our research believed that STIs are a punishment from God, and generally believed that people with an STI deserved it [5]. Negative views towards people living with HIV/AIDS are highly prevalent among Muslims both in the community and among healthcare workers [31]. This strong stigma and taboos towards HIV/AIDS have been consistently reported across the MENA region [32]. Stigma and discrimination in healthcare settings is also evident in Islamic countries [31]. Medical students from Saudi Arabia reported extremely negative views towards people living with HIV/AIDS [33]. In a study in Iran on people living with HIV/AIDS, all study participants, with no exception, reported experiencing denial of care from healthcare providers due to their negative views towards drug use, sex work, and extramarital sex [31].

Poor knowledge of STI is common among Muslims worldwide [5–7, 19, 20]. Poor knowledge can contribute to stigma and discrimination against people living with HIV/AIDS [34]. For example, physicians in Saudi Arabia who had poor knowledge of HIV/AIDS had significantly higher mean stigma scores than those who had good knowledge [10].

Consistent with our findings, HIV/AIDS is the most widely recognised STI globally, mainly due to the extensive awareness campaigns taking place since the mid 1980s [35]. This highlights the importance of national and international awareness campaigns in improving health literacy and promoting health behaviours. Although improved sexual health knowledge is not sufficient by itself to promote protective health behaviours, it has the potential to promote safer sexual practices, condom use, and improve attitudes towards STIs that are necessary for timely testing and diagnosis [36]. An urgent need for education was expressed by research participants, to raise awareness and prevent future STI transmissions. Nearly 86% of adolescents in Saudi Arabia called for the need for school-based sex education, with 92.2% believing that sex education is required to protect against STIs and establish healthy sexual behaviours [21]. Research and public health efforts should be directed towards improving STIs awareness in Saudi Arabia and facilitating protective health behaviours.

Women in this research thought of men as the source of most STIs, with women usually viewed as the victims. The majority of infections among married women in the MENA region are from the husband, and marriage is considered a risk factor for many women [13–16]. Nearly 97% of women living with HIV in Saudi Arabia [37], 76% in Iran [38], and 70% in Morocco acquired it from their spouse [13]. Husbands could either knowingly or unknowingly transmit the infection to their wives [14, 26]. Women generally lack the power to negotiate safe sex with their partners or guarantee their faithfulness [14]. In Egypt for example, women infected with HIV from spouses explained that they were aware of their husbands’ risk behaviours but felt helpless and unable to protect themselves, mainly due to lack of sexual autonomy and inability to influence their husbands’ high risk behaviours [39]. Religious, economic, and societal factors combined with the imbalance of power in a marital/sexual relationship all contribute to women’s vulnerability to STIs [31].

As a result of the normalised discrimination against women, the social implications of STIs are much more severe for women regardless of the route of transmission [26]. This gendered discrimination has been consistently reported, and women are subjected to more judgement and blame than men [5]. The social consequences of premarital sex are much more severe and dangerous for Muslim women [40]. Premarital sex, more specifically losing virginity or unintentional pregnancy, is one of the leading causes of suicide and honour killings for young Muslim women worldwide [40]. For example, in Algeria, 30% of women who committed suicide were pregnant outside of marriage [13]. In Turkey, hymen examinations (i.e., questioning virginity) are the most common cause of suicide among young Turkish women [13]. Many honour killings go unpunished, and deaths are concealed as suicides or accidents [41].

## Strengths and limitations

To our knowledge, this is the first qualitative study to explore STI knowledge, views, and attitudes in Saudi Arabia. Our findings are transferable to women in Muslim communities around the world, as many Muslims share similar cultural values and traditions [42–45]. Interviewing women from different age groups and marital status allowed for comparisons to be made across different experiences, offering an opportunity to explore the narratives of unmarried women. The lead author is a young, Saudi female experienced in qualitative research. This could have facilitated the recruitment and the interview process through shared language and cultural references.

We attempted to reduce social desirability bias by carefully wording questions to avoid leading questions, trying not to show judgment, and remaining neutral towards participants’ responses. This research took place in Riyadh, the capital city of Saudi Arabia. The residents of Riyadh city are more likely to be liberal and highly educated. Therefore, the views of women who are less educated, more conservative, and less likely to be willing to discuss sexual health topics may be underrepresented.

## Research and policy implications

An important implication from our findings is reducing the specific vulnerability of Muslim women to STIs. Women need to be informed about STIs symptoms, modes of transmission, and prevention in order to recognise the signs of STIs and provide them with the tools to protect themselves. This is especially important for women who are aware of their partners’ behaviours but uninformed on ways to protect themselves. More importantly, strategies to improve women’s empowerment and sexual autonomy are highly needed in the region.

Since HIV risk in the MENA among the general population is low yet considerable among priority groups (e.g., sex workers, injecting drug users), programmes directed towards the general population should stress stigma reduction, rather than personal risk reduction. Public health efforts should be directed towards reducing the stigma and discrimination against people living with HIV/AIDS among Muslim communities.

Research efforts should focus on exploring barriers to STIs testing and diagnosis among Muslim communities and explore how to tackle stigma and discrimination against people living with HIV/AIDS.

Our research did not include men’s perspectives. future research should explore Muslim men’s views and experiences to gain a comprehensive understanding of sexual health issues in Muslim communities.

## Conclusion

Muslim women’s attitudes towards STIs, especially HIV/AIDS, were highly influenced by Islamic views forbidding extramarital sex, as well as lack of knowledge and misconceptions. There is an urgent need for the formulation and dissemination of accurate sexual health information including STI symptoms, modes of transmission, prevention, and treatment. Public health efforts should be directed towards reducing the stigma and discrimination against people living with HIV/AIDS in Saudi Arabia and other Islamic communities.
